# The Mechanism of Functional Up-Regulation of P2X3 Receptors of Trigeminal Sensory Neurons in a Genetic Mouse Model of Familial Hemiplegic Migraine Type 1 (FHM-1)

**DOI:** 10.1371/journal.pone.0060677

**Published:** 2013-04-05

**Authors:** Swathi K. Hullugundi, Michel D. Ferrari, Arn M. J. M. van den Maagdenberg, Andrea Nistri

**Affiliations:** 1 Neuroscience Department, International School for Advanced Studies (SISSA),Trieste, Italy; 2 Department of Neurology, Leiden University Medical Centre, Leiden, The Netherlands; 3 Department of Human Genetics, Leiden Genetics University Medical Centre, Leiden, The Netherlands; Dalhousie University, Canada

## Abstract

A knock-in (KI) mouse model of FHM-1 expressing the R192Q missense mutation of the C*acna1a* gene coding for the α1 subunit of Ca_V_2.1 channels shows, at the level of the trigeminal ganglion, selective functional up-regulation of ATP -gated P2X3 receptors of sensory neurons that convey nociceptive signals to the brainstem. Why P2X3 receptors are constitutively more responsive, however, remains unclear as their membrane expression and TRPV1 nociceptor activity are the same as in wildtype (WT) neurons. Using primary cultures of WT or KI trigeminal ganglia, we investigated whether soluble compounds that may contribute to initiating (or maintaining) migraine attacks, such as TNFα, CGRP, and BDNF, might be responsible for increasing P2X3 receptor responses. Exogenous application of TNFα potentiated P2X3 receptor-mediated currents of WT but not of KI neurons, most of which expressed both the P2X3 receptor and the TNFα receptor TNFR2. However, sustained TNFα neutralization failed to change WT or KI P2X3 receptor currents. This suggests that endogenous TNFα does not regulate P2X3 receptor responses. Nonetheless, on cultures made from both genotypes, exogenous TNFα enhanced TRPV1 receptor-mediated currents expressed by a few neurons, suggesting transient amplification of TRPV1 nociceptor responses. CGRP increased P2X3 receptor currents only in WT cultures, although prolonged CGRP receptor antagonism or BDNF neutralization reduced KI currents to WT levels. Our data suggest that, in KI trigeminal ganglion cultures, constitutive up-regulation of P2X3 receptors probably is already maximal and is apparently contributed by basal CGRP and BDNF levels, thereby rendering these neurons more responsive to extracellular ATP.

## Introduction

Familial Hemiplegic Migraine type 1 (FHM-1) is a rare monogenic subtype of migraine with aura that is caused by missense mutations in the *CACNA1A* gene [1] that encodes the α1 subunit of neuronal voltage-gated Ca_V_2.1(voltage-gated calcium channel type 2.1) calcium channels [2]. Knock-in (KI) mice that express FHM-1 R192Q-mutated Ca_V_2.1 channels reveal gain-of-function effects on Ca_V_2.1 channels that can explain the increased susceptibility to cortical spreading depression [3], [4], the likely cause of the migraine aura [5]. At the level of trigeminal sensory ganglion neurons, which code nociceptive signals to be sent to the brainstem trigeminal complex, the FHM-1 R192Q KI mice show a selective gain-of-function phenotype on ATP (adenosine-5-triphosphate)-gated P2X3 (purinergic ionotropic receptor 3) receptors [6]. Indeed, an upward shift in the agonist concentration/response curve was demonstrated, without a change in membrane receptor expression or of other ionotropic receptors such as TRPV1 (transient receptor potential vanilloid 1) receptors. Our previous experiments indicated that P2X3 receptor up-regulation is brought about by an altered phosphorylation state of the intracellular P2X3 receptor domains [6]. However, this does not explain the primary cause for the observed enhancement of P2X3 receptor responses. As P2X3 receptors in the brain and spinal cord are almost exclusively expressed on sensory neurons that transfer nociceptive signals to higher centers [7], [8], unraveling the primary mechanism may have significant relevance to understanding the generation of migraine-relevant pain processes. Since extracellular ATP levels are usually low [9], P2X3 receptors are not continuously activated under basal conditions in WT sensory neurons. However, constitutively up-regulated P2X3 receptors as seen in R192Q KI sensory neurons, are responsive already after application of low doses of the selective agonist α,β-methyleneadenosine 5-triphosphate (α,β-meATP) [6]. Hence, this mutant P2X3 receptor phenotype may bring about a selective rise in receptor efficacy in a migraine mouse model.

Here we investigated whether soluble factors (“migraine mediators”; [10]), i.e. CGRP (calcitonin gene related peptide), BDNF (brain derived neurotrophin factor) or TNFα (tumor necrosis factor alpha), which are relevant to triggering migraine attacks [11], [12], [13], [14], [15], [16], [17], might constitutively potentiate P2X3 receptor currents. Already a stronger release of CGRP and TNFα was reported in R192Q KI trigeminal ganglia [18], [19], [20]. It is also noteworthy that a substantial part of the action of CGRP is produced via release of endogenous BDNF [21] and that CGRP and BDNF may synergize to induce pain mechanisms at trigeminal ganglion level [22], [23].

The aim of the present report is to determine how “migraine mediators” shape P2X3 receptor activity of cultured R192Q KI trigeminal ganglion neurons. We applied the CGRP-selective antagonist CGRP _8–37_, or introduced an overnight deprivation of BDNF or TNFα to dissect the effects of their endogenous concentrations on P2X3 receptor responses. In addition, we applied exogenous CGRP or TNFα to the cultures to investigate if R192Q KI neurons could show the same degree of P2X3 receptor potentiation that is usually found for WT neurons [24], [25].

## Results

### TNFα receptor expression and activation on trigeminal neurons

**Figure 1:**
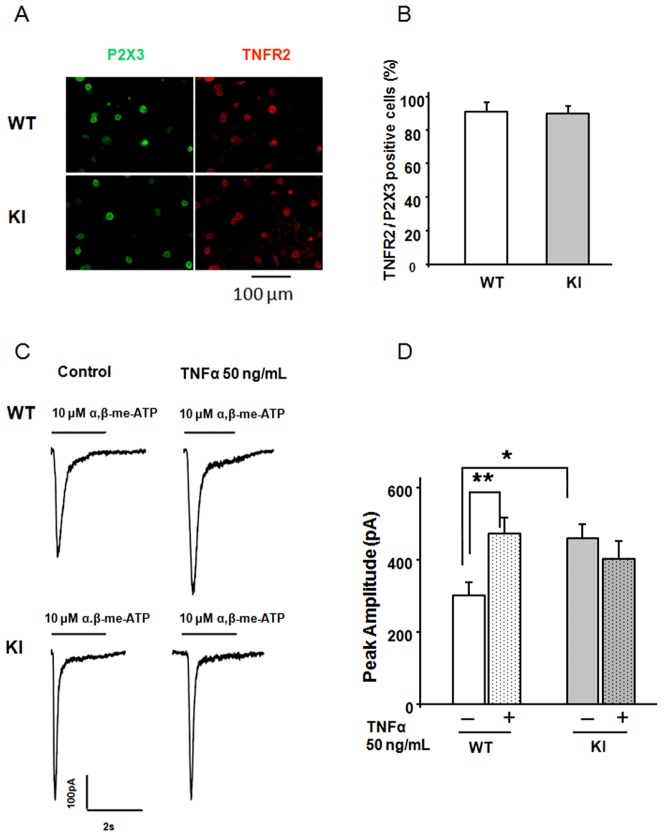
Effect of TNFα on P2X3 receptor activity and co-expression of its TNFR2 receptors. A, Examples of TNFR2 and P2X3 co-exexpression in (wildtype) WT and R192Q (knockin) KI neurons. Left panel shows P2X3 expression (green), and right panel shows TNFR2 staining (red). B, Histograms quantifying % of cells co-expressing TNFR2 and P2X3: both WT and KI cultures show similar TNFR2 and P2X3 co-expression. N = 3 independent experiments (6 mice). C, Representative traces of currents induced by application of α,β-meATP (10 µM, 2 s) to WT or R192Q KI neurons in control conditions or after 4 h TNFα application. D, Histograms show average peak amplitudes of P2X3 receptor-mediated currents: WT control (open bar), n = 30; WT TNFα (stippled bar), n = 38; KI control (grey bar), n = 34; KI TNFα (stippled gray bar), n = 34; ** = p<0.006; * = p<0.05.

Sensory ganglia, including trigeminal ganglia, from rats were previously shown to synthetize and release the inflammatory mediator TNFα [18], [26], [27]. As recently reviewed [28], [29], the cellular effects of TNFα are mediated by two distinct receptor classes (TNFR1 and TNFR2).While TNFR1 is ubiquitously expressed and is canonically considered to be a pro-apoptotic signal in cell death pathways, TNFR2 expression is restricted to immune cells like microglia and macrophages, neurons and oligodendrocytes. In view of previous reports that neuroinflammation might be contributory to sensitize neurons in migraine attacks [9], [18], [30], [31], we first investigated whether mouse trigeminal ganglion neurons could respond to TNFα as evidenced by their expression of the TNFα receptor TNFR2 ([32]; Fig. 1A, B). The large majority of trigeminal ganglion neurons in both WT and KI cultures were also immunopositive for P2X3 antibody (Fig. 1A, B). This observation suggests that mouse trigeminal ganglion neurons could be a target for TNFα, an issue investigated by whole-cell patch clamping of neurons in response to pulses of P2X3 receptor agonist α,β-meATP (Fig. 1C). Fast inward currents elicited by α,β-meATP (10 µM; 2 s) on WT neurons were enhanced by a 4-hour pretreatment with TNFα (50 ng/mL; Fig. 1 C,D). A similar increase in current amplitude was observed with 100 ng/mL TNFα (406±49 pA versus 282±35 pA of untreated control, n = 10 and n = 14, respectively; p = 0.04). Notably, P2X3 receptor currents of KI neurons, which are constitutively larger than of WT ones (Fig. 1D and [6]), were not further potentiated by 50 ng/mL TNFα pretreatment (Fig. 1 C, D). Even using 100 ng/mL TNFα, the average peak amplitude of treated KI neurons was 389±48 pA (n = 10); a value not significantly different from untreated control (438±70 pA, n = 11). These data are in accordance with our previous results [18] that indicated how experimentally-evoked increase in TNFα gene expression was followed by facilitation of P2X3 receptors in WT neurons only.

**Figure 2:**
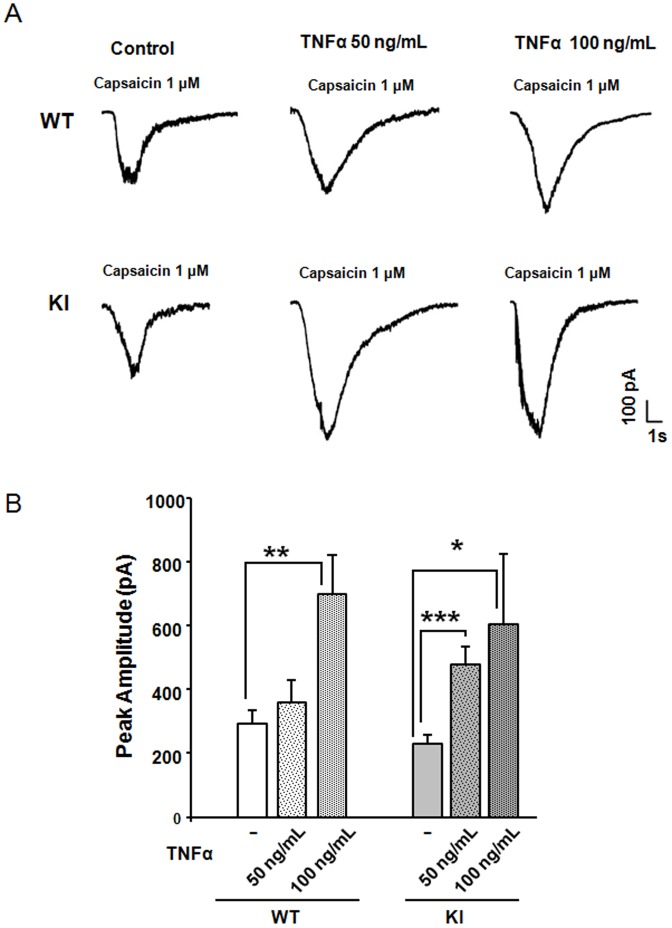
Sensitization of TRPV1 receptors by TNFα. A, Representative traces of currents induced by application of capsaicin (1 µM, 2 s) to WT or R192Q KI neurons in control conditions or after 4 h application of TNFα. B, Histograms show average peak amplitudes of TRPV1-mediated currents (WT control, n = 29; WT TNFα 50 ng/mL n = 19, WT TNFα 100 ng/mL, n = 10; KI control, n = 26; KI TNFα 50 ng/mL, n = 23,, KI TNFα 100 ng/mL n = 9); * = p<0.05, ** = p<0.002, *** = p<0.001.

Former studies have indicated that exogenous TNFα can increase neuronal Ca^2+^ influx via augmented activity of TRPV1 receptors on rat sensory neurons [33], [34]. We investigated whether TNFα application to mouse trigeminal ganglion cultures could also potentiate membrane TRPV1 receptor currents that were activated by pulses of capsaicin (1 µM; 2 s), as reported for rat neurons [35], [36]. Fig. 2A shows sample traces of enhancement of capsaicin-evoked currents by prior application of TNFα on small diameter neurons, which typically express TRPV1 receptors [6], [37]. It is noteworthy that, on average, WT and KI TRPV1 currents had similar amplitudes under control conditions (Fig. 2 B), indicating that there was no phenotype-dependent TRPV1 regulation. While TRPV1 receptors on WT neurons required a large dose of TNFα to exhibit potentiation, responses of KI neurons required only half of that dose (Fig. 2B). These results, therefore, suggest that TRPV1 receptors of KI neurons were more susceptible to transient application of TNFα.

**Figure 3:**
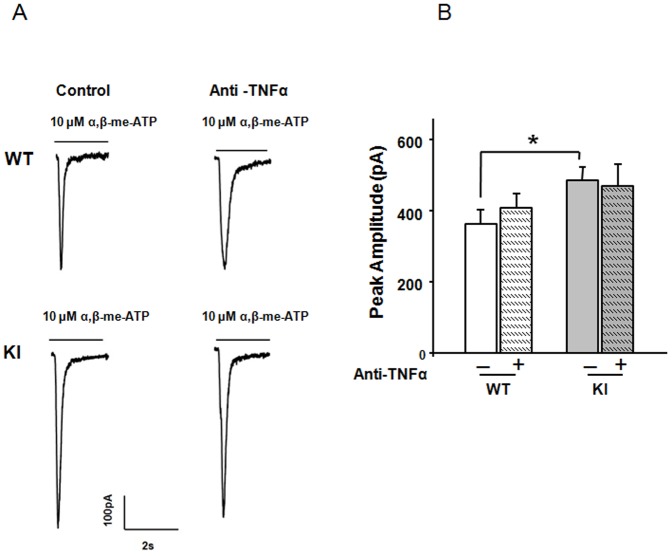
Ambient TNFα may not contribute to basal upregulation of KI P2X3 receptor function. A, Representative traces of currents induced by application of α,β-meATP (10 µM, 2 s) to WT or R192Q KI neurons in control conditions or after overnight application of anti-TNFα antibody. B, Histograms show average peak amplitudes of P2X3 receptor-mediated currents (WT control, n = 23; WT anti-TNFα, n = 14; KI control, n = 34; KI anti-TNFα, n = 20). * = p<0.05.

We next enquired whether basal, strong responses of P2X3 receptors of KI neurons might be due to a constitutively high release of endogenous TNFα, which would prevent further increment of P2X3 receptor-mediated responses. To investigate this, we caused TNFα deprivation by overnight administering a TNFα neutralizing antibody, and, after washout, recorded responses to α,β-meATP. A similar deprivation protocol had been used to investigate the role of endogenous NGF, another potent algogen on trigeminal ganglion neurons [38]. The TNFα deprivation protocol did not change P2X3 receptor responses in either WT or KI neurons (Fig. 3A, B). The simplest interpretation of these results is that there was insufficient background release of endogenous TNFα to modulate P2X3 receptors under basal conditions. This notion prompted us to investigate other mechanisms that might be responsible for the P2X3 receptor enhancement that was observed in R192Q KI neurons.

### Effect of CGRP antagonism or BDNF deprivation on P2X3 receptors

**Figure 4:**
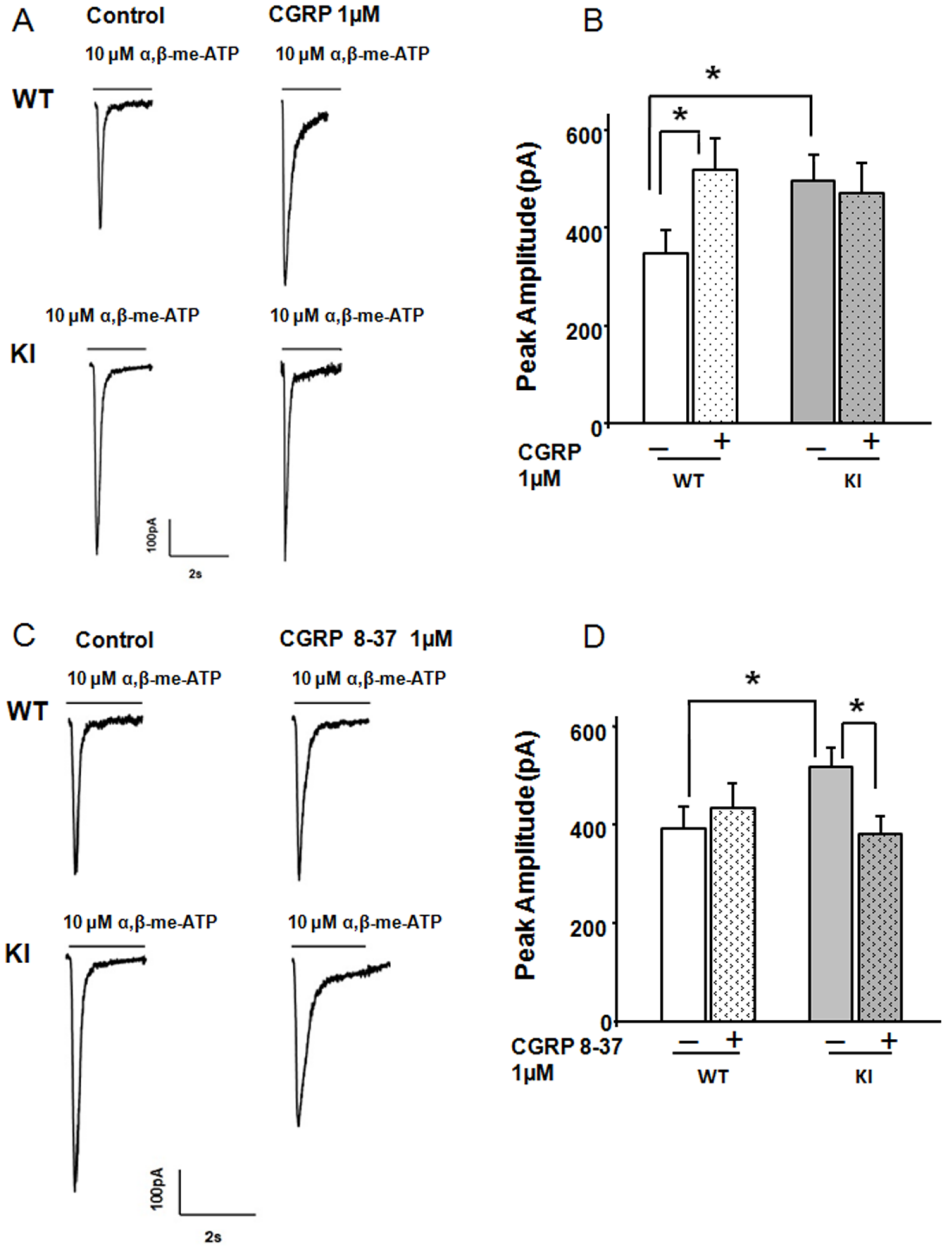
Role of CGRP in KI P2X3 currents. A, Representative traces of currents induced by application of α,β-meATP (10 µM, 2 s) to WT or R192Q KI neurons in control conditions or after 2 h CGRP (1 µM) application. B, Histograms show average peak amplitudes of P2X3 receptor-mediated currents (WT control, n = 11; WT CGRP, n = 9; KI control, n = 17; KI CGRP, n = 18), * = p<0.05. C, Representative examples of currents induced by application of α,β-meATP (10 µM, 2 s) to WT or R192Q KI neurons in control conditions or after overnight application of the CGRP antagonist CGRP_8–37_ (1 µM). D, Histograms show average peak amplitudes of P2X3 receptor-mediated currents (WT control, n = 23; WT CGRP_8–37_, n = 23; KI control, n = 29; KI CGRP_8–37_, n = 30); * = p<0.05.

Next, we investigated the action of the neuropeptide CGRP, an important migraine mediator that is known to evoke a sustained facilitation of P2X3 receptors via complex intracellular signaling pathways in WT trigeminal ganglia [19], [24]. A role of CGRP in KI trigeminal ganglia, however, is less obvious. Previously, R192Q KI ganglia were shown to contain less CGRP than WT [39], an observation that may be accounted for by a stronger release of CGRP [19]. Fig. 4A, B shows that CGRP (1 µM, 2 h; [24]) significantly enhanced WT P2X3 responses without affecting those of KI. One may postulate that potentiation of P2X3 receptors could be already at the maximal level, because of raised endogenous CGRP, and, therefore, these receptors had become unresponsive to further CGRP administration. Measuring CGRP concentrations in the bulk culture medium or in culture extracts may not be suitable for providing the actual concentration at neuronal level because of large dilution and/or degradation the peptide. Therefore, we investigated the influence of constitutive release of CGRP on P2X3 receptors, by overnight application of the specific CGRP receptor antagonist CGRP_8–37_ [24], [40]. Fig. 4C, D illustrates that CGRP_8–37_ pretreatment did not change the amplitude of P2X3 receptor currents of WT neurons, but instead returned P2X3 responses of KI neurons to the same level of WT neurons. These data point to a significant role of endogenous CGRP in facilitating P2X3 receptor currents, specifically of KI neurons.

**Figure 5:**
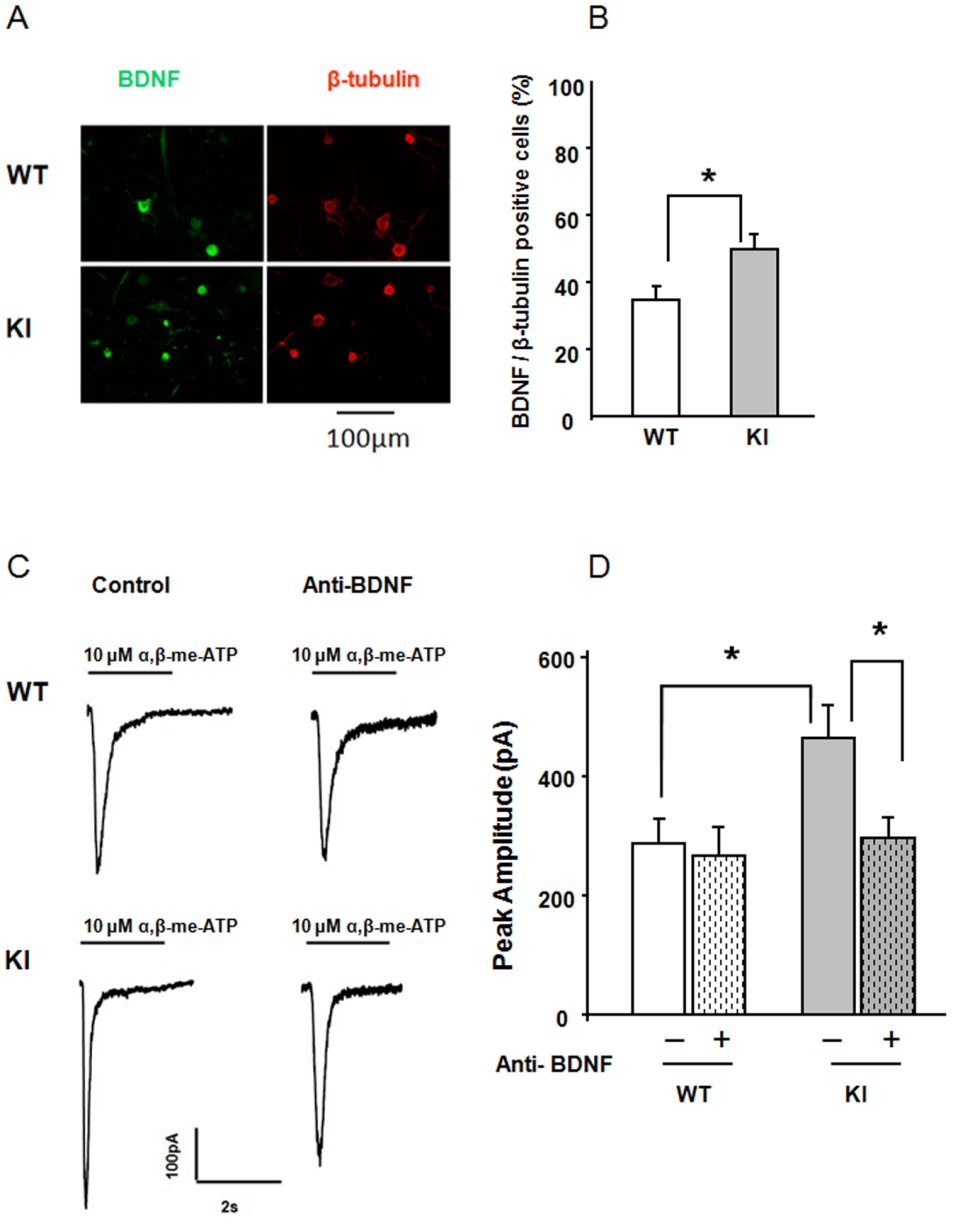
Number of BDNF expressing neurons in KI culture and effect of BDNF deprivation. A, Examples of β-tubulin positive neurons expressing BDNF in WT or KI cultures. Left panel (green) shows BDNF expression and right panel (red) shows β-tubulin staining of the same neurons. B, Histograms quantifying % of neurons expressing BDNF: KI cultures showed significantly higher number of BDNF positive neurons. N = 4 independent experiments (8 mice), p<0.05. C, Representative traces of currents induced by application of α,β-meATP (10 µM, 2 s) to WT or R192Q KI neurons in control conditions or after overnight application of anti-BDNF antibody. D, Histograms show average peak amplitudes of P2X3 receptor-mediated currents (WT control, n = 9; WT anti-BDNF, n = 10; KI control, n = 32; KI anti-BDNF, n = 38); * = p<0.05.

Previous studies [21] have demonstrated that a substantial component of the action by CGRP on trigeminal ganglion neurons is caused indirectly via release of BDNF, a known algogen [13], [21], [41], [42,]. We queried whether the basal potentiation of P2X3 receptors of KI neurons was also due to the combined action of CGRP and BDNF. To this end, first we confirmed that BDNF significantly (p = 0.019) potentiated WT P2X3 receptors as the average peak current amplitude rose from 281±31 pA (n = 15) to 464±61 pA (n = 18) in support of our earlier reports which have shown increased P2X3 mRNA upon BDNF treatment [21]. Second, we assessed the BDNF immunoreactivity of trigeminal ganglion neurons of WT or KI cultures (Fig. 5A, B). The average number of BDNF-positive neurons (identified by β-tubulin III immunopositive signals; Fig. 5A, red pseudocolor) was significantly higher in KI cultures compared to WT cultures. In support of this finding, overnight treatment with a BDNF neutralizing antibody [21] showed no change in P2X3 receptor currents of WT neurons, whereas the current amplitude of KI neurons was significantly decreased (Fig. 5C, D).

## Discussion

The present study proposes that basal release of CGRP and BDNF contributes to the observed potentiation of P2X3 receptors of FHM-1 R192Q KI trigeminal neurons. It seems probable that facilitated release of CGRP and BDNF, at least in part, was dependent on the stronger Ca^2+^ influx into R192Q KI neurons [6], which is in line with other gain-of-function effects of the R192Q-mutated Ca_V_2.1 channels as reported for brainstem [43] and cerebral cortex [4] excitatory synapses.

Since CGRP is considered an important trigger of migraine attacks, current clinical studies have focused on the use of CGRP antagonists as a possible treatment [16], [17], [44], [45]. The involvement of CGRP mechanisms in the pathophysiology of FHM has been debated, for instance by reports showing lack of hypersensitivity of FHM patients to CGRP infusion [46], [47]. However, our findings argue that, in trigeminal neurons expressing the R192Q mutation in Ca_V_2.1 channels (as in R192Q KI mice), potentiation of P2X3 receptors was no longer possible by exogenous CGRP as this effect had reached saturation. A role of endogenous CGRP is also supported by the observation that overnight application of a selective CGRP antagonist could return P2X3 response level in KI neurons to that of WT neurons. Hence, we propose that trigeminal neurons expressing the R192Q FHM-1 mutation are already enhanced in their P2X3 receptor sensitivity by endogenously elevated CGRP levels, and, therefore, cannot be further potentiated by the exogenously administered CGRP peptide.

Blocking the action of endogenous CGRP or BDNF (an intermediate mediator of the effect by CGRP on P2X3 receptors; [21]) led to significant reduction of P2X3 currents to WT levels. In WT neurons, 50% of the potentiation of P2X3 receptors by CGRP is attributed to release of endogenous BDNF [21]. In R192Q KI trigeminal ganglia, the role of BDNF in P2X3 receptor response likely is relevant as the majority of P2X3-positive neurons express this neurotrophin. In view of the synergy of BDNF and glutamate (the excitatory transmitter of trigeminal ganglion neurons) in synaptic transmission [48], [49], if enhanced release of BDNF occurs in the brain as well, it seems plausible that this neurotrophin contributes to the dysregulated neuronal excitability caused by overactive glutamatergic synapses of R192Q-mutated cortical neurons [4].

Clinical and experimental studies have proposed that a neuroinflammatory state of meninges, enriched with inflammatory cells releasing pain mediators like TNFα [50], known to play an important role in chronic pain [51], might contribute to migraine attacks [30], [31]. We recently provided first evidence for a proinflammatory profile, both *in vivo* and *in vitro*, in R192Q KI trigeminal ganglia, which suggests that neuroinflammation may occur also in this migraine mouse model [26]. Here, we could show that, in WT or KI trigeminal ganglia, the large majority of neurons co-express P2X3 and TNFα receptors rendering them potentially sensitive to TNFα-mediated inflammation and related pain signaling. Nevertheless, the phenomenon of exogenous TNFα enhanced P2X3 responses exclusively in WT neurons and not in KI cells, is analogous to the one seen with exogenous CGRP and is probably caused by P2X3 receptor saturation. Lack of exogenous TNFα effect on R192Q KI neurons was not attributable to rapid degradation or unresponsive receptors because the same substance readily facilitated TRPV1 receptors. The latter observation is in accordance with previous reports with WT sensory cells [35], [36] and it was obtained after a smaller dose of TNFα for KI neurons. Future *in vivo* experiments are necessary to assess whether the TNFα-evoked potentiation of TRPV1 currents plays a major functional role in processing nociceptive signals as these receptors are expressed by a minority of neurons (usually half of the small diameter cells; [20], [37] and, under basal conditions, they do not functionally differ between WT and KI neurons [6].

Unlike the observations with CGRP and BDNF, an overnight deprivation of TNFα was not followed by down-regulation of P2X3 receptors in WT or KI neurons, which may be due to the low level of endogenous TNFα [18]. Furthermore, while previous studies have indicated a close, positive interaction between TNFα and CGRP [52], this effect was presumably weak in trigeminal ganglion cultures as the functional outcome of overnight deprivation of each substance was different. One attractive hypothesis that deserves further attention is that, in R192Q KI trigeminal ganglia, enhanced endogenous CGRP and BDNF levels contribute to up-regulate basal P2X3 receptor function of most sensory neurons, preparing them to respond more efficiently to extracellular ATP, a putative mediator of migraine attacks [53]. On the other hand, a surge in extracellular TNFα might serve to increase TRPV1 receptor activity and, thus, perhaps amplify peripheral nociceptive transduction which is thought to be important for migraine pain [54].

## Materials and Methods

### Culture of mouse trigeminal ganglia

For the experiments, R192Q KI and WT mouse littermates were used. Our colony of mice is bred locally, after an initial transfer of mice from Leiden University Medical Centre [3]. Mice were maintained in accordance with the Italian Animal Welfare Act. The experimental protocols were approved by the SISSA ethical committee. Genotyping was performed by polymerase chain reaction (PCR), as previously reported [3]. Trigeminal ganglion cultures were obtained from 2-week-old animals and were used 24 h after plating [6], [21]. For all experiments, WT and KI cultures were used in parallel at the same time to allow for direct comparison of experimental data.

### Immunostaining

Primary cultures obtained from two ganglia were plated on 2–3 coverslips for parallel analysis of immunoreactive signals. Cultures were fixed with 4% PFA and processed for immunofluorescence microscopy [6], [21]. The following antibodies, whose selectivity has been validated previously [6], [21], were used: anti-P2X3 (1∶300; Neuromics, Edina, MN, USA), anti-TNFR2 (1∶50, Santacruz Biotechnology, Heildelberg, Germany), anti-β-tubulin III (1∶1000; Sigma, Milan, Italy); anti-BDNF (1∶50; Sigma). Antibodies were incubated for 2 h in phosphate saline buffer plus 5% bovine serum albumin and 0.1% Tween20. Secondary antibodies anti-rabbit, anti- conjugated with AlexaFluor488 or AlexaFluor594 were purchased from Invitrogen (1∶500; S. Giuliano Milanese, Italy). Stainings with secondary antibodies only were performed as control experiments and did not give any signal. Images from cultures were visualized with a Zeiss Axioskop fluorescence microscope (Zeiss, Zurich, Switzerland) and analyzed with MetaMorph software (Molecular Devices, Downingtown, PA, USA) for cell counting. The number of neurons expressing TNFR2 was calculated as a percent value of the total number of neurons expressing P2X3 receptors. BDNF-immunoreactive neurons were expressed as percent value of β-tubulin-positive neurons. Data are the mean of at least three independent experiments.

### Electrophysiology

After 1 day in culture, trigeminal neurons were superfused continuously (2 mL/min) with physiological solution containing (in mM): 152 NaCl, 5 KCl, 1 MgCl_2_, 2 CaCl_2_, 10 glucose, and 10 HEPES (pH adjusted to 7.4 with NaOH). Cells were patch-clamped in the whole-cell configuration using pipettes with a resistance of 3–4 MΩ when filled with the following solution (in mM): 140 KCl, 0.5 CaCl_2_, 2 MgCl_2_, 2 Mg_2_ATP_3_, 2 GTP, 10 HEPES, and 10 EGTA (pH adjusted to 7.2 with KOH). Recording of P2X3 receptor-mediated currents was performed mostly on small and medium size neurons with capacitance less than 20 pF (ranging from 8 to 23 pF), the vast majority of which expresses these receptors [35]. For recording capsaicin-induced currents, data were obtained from small diameter neurons (capacity<15 pF). Cells were held at −65 mV after correcting for the liquid junction potential. Currents were filtered at 1 kHz and acquired by means of a DigiData 1200 Interface and pClamp 8.2 software (Molecular Devices, Sunnyvale, CA, USA). To obtain stable and reproducible P2X3 receptor currents, its synthetic agonist α,β-methylene-adenosine-5′-triphosphate (α,β-meATP; 10 µM; Sigma) was applied (for 2 s) using a fast superfusion system (Rapid Solution Changer RSC-200; BioLogic Science Instruments, Claix, France). In accordance with our previous studies [6], [9], [18], this concentration of α,β-meATP was selected as it evokes near maximal responses that were suitable to test any depression caused by prolonged deprivation of endogeneous factors. In line with previous reports with the same type of WT or KI neurons [6], [20], capsaicin (Sigma) was applied at the concentration of 1 µM (for 2 s) to evoke reproducible inward currents. In all experiments employing overnight or prolonged treatment of culture, the culture medium was washed out with physiological solution at the end of the incubation period and patch clamp recording was commenced within 10 min. For deprivation experiments the following treatments were applied for 24 h starting at the time of plating the cultures: anti-BDNF (neutralizing antibody; final dose = 10 µg/mL; Promega, Madison, WI, USA), or anti-TNF-α (neutralizing antibody; 1∶200 from 5 mg/mL stock; Abcam, Cambridge, UK). The neutralizing TNFα antibody and the BDNF antibodies were validated with Western immunoblotting for their effectiveness [18], [21]. The CGRP antagonist peptide CGRP_8–37_ (Sigma) was applied overnight at 1 µM concentration [24]. TNFα (R&D microsystems, Abingdon, UK) was applied at the dose of 50 or 100 ng/mL as previously performed for sensory ganglia [41], [52]. The timing of application of TNFα (4 h) was chosen based on the observation that its endogenous production and activity on trigeminal ganglion neurons peak 4 h after experimental inflammation [18]. CGRP (1 µM; Sigma) or BDNF (10 ng/mL; Sigma) were applied for 2 h as was done in a previous study [21], [24].

### Statistics

Data are expressed as mean ± SEM (standard error of the mean), where n indicates the number of independent experiments or the number of investigated cells. Statistical analysis was performed using the Student's t test or the Mann–Whitney rank sum test after the software-directed choice of parametric or nonparametric data, respectively (Sigma Plot and Sigma Stat, Chicago, IL,USA). A p value of ≤0.05 was accepted as indicative of a statistically significant difference.
